# Light and electron microscopy characteristics of the muscle of patients with *SURF1* gene mutations associated with Leigh disease

**DOI:** 10.1136/jcp.2007.051060

**Published:** 2007-10-01

**Authors:** M Pronicki, E Matyja, D Piekutowska-Abramczuk, T Szymańska-Dębińska, A Karkucińska-Więckowska, E Karczmarewicz, W Grajkowska, T Kmieć, E Popowska, J Sykut-Cegielska

**Affiliations:** 1 Department of Pathology, The Children’s Memorial Health Institute, Warsaw, Poland; 2 Department of Metabolic Diseases, Endocrinology and Diabetology, The Children’s Memorial Health Institute, Warsaw, Poland; 3 Department of Medical Genetics, The Children’s Memorial Health Institute, Warsaw, Poland; 4 Department of Biochemistry and Experimental Medicine, The Children’s Memorial Health Institute, Warsaw, Poland; 5 Department of Neurology and Epileptology, The Children’s Memorial Health Institute, Warsaw, Poland; 6 Department of Experimental and Clinical Neuropathology, M. Mossakowski Medical Research Centre, Polish Academy of Sciences, Warsaw, Poland

## Abstract

**Aims:**

Leigh syndrome (LS) is characterised by almost identical brain changes despite considerable causal heterogeneity. *SURF1* gene mutations are among the most frequent causes of LS. Although deficiency of cytochrome *c* oxidase (COX) is a typical feature of the muscle in SURF1-deficient LS, other abnormalities have been rarely described. The aim of the present work is to assess the skeletal muscle morphology coexisting with *SURF1* mutations from our own research and in the literature.

**Methods:**

Muscle samples from 21 patients who fulfilled the criteria of LS and *SURF1* mutations (14 homozygotes and 7 heterozygotes of c.841delCT) were examined by light and electron microscopy.

**Results:**

Diffuse decreased activity or total deficit of COX was revealed histochemically in all examined muscles. No ragged red fibres (RRFs) were seen. Lipid accumulation and fibre size variability were found in 14 and 9 specimens, respectively. Ultrastructural assessment showed several mitochondrial abnormalities, lipid deposits, myofibrillar disorganisation and other minor changes. In five cases no ultrastructural changes were found. Apart from slight correlation between lipid accumulation shown by histochemical and ultrastructural techniques, no other correlations were revealed between parameters investigated, especially between severity of morphological changes and the patient’s age at the biopsy.

**Conclusion:**

Histological and histochemical features of muscle of genetically homogenous SURF1-deficient LS were reproducible in detection of COX deficit. Minor muscle changes were not commonly present. Also, ultrastructural abnormalities were not a consistent feature. It should be emphasised that SURF1-deficient muscle assessed in the light and electron microscopy panel may be interpreted as normal if COX staining is not employed.

Leigh syndrome (LS), first described in 1951, is a severe neurodegenerative disease with characteristic neuropathological lesions located mainly in the basal ganglia, brain stem and sometimes other structures of central nervous system.^1^ Since the first description, hundreds of patients with LS-type brain changes have been reported. Various respiratory chain dysfunctions and/or the presence of different mitochondrial DNA mutations have been considered characteristic for all LS patients.^2^

The *SURF1* gene discovered in 1998 is the first nuclear gene for which the association of its mutations with deficiency of respiratory chain complex IV (cytochrome *c* oxidase (COX)) has been confirmed in humans.^3^ ^4^ The *SURF1* mutations were found invariably and almost exclusively in patients with generalised COX-deficit coexistent with LS features. Nearly 60 such patients and over 35 different mutations have been reported so far in the literature.^5^^–^32 *SURF1* encodes for one of several proteins involved in proper assembly of the active COX complex.

Despite numerous detailed reports in muscle pathology in many different mitochondrial disorders^33^ ^34^ the character of skeletal muscle lesions in SURF1-deficient LS has not been precisely described to date. In order to establish if there are any lesions of skeletal muscle specific for the *SURF1* gene mutated patients, we summarised results of muscle biopsy in 21 patients with confirmed *SURF1* gene mutations. Our analysis also includes data on 15 patients available from the literature (patients were of English, French and Czech nationality).

## PATIENTS AND METHODS

Retrospective pathological investigations were performed in all children with the diagnosis of LS established at our mitochondrial centre during the period 1994–2005 in whom genetic molecular studies eventually revealed the presence of *SURF1* gene mutations.

Skeletal muscle samples from 21 patients from 20 unrelated Polish families (11 boys and 10 girls, aged from 9 months to 12 years) were reassessed by light and/or electron microscopy.

Commonly accepted clinical criteria was applied for establishing the LS diagnosis in the patients.^35^ Biochemical assays of muscle homogenates showed a decrease in activity of complex IV of the respiratory chain and an increase in activity of citric synthase (CS). Molecular analysis of the *SURF1* gene revealed the presence of the c.841delCT (formerly c.845delCT) mutation in all patients studied. Twelve patients were homozygous for this mutation, and the remaining seven were compound heterozygous. Clinical, biochemical and molecular characteristics of the patients are presented in table 1, and were also published in part previously.^13^ ^27^ ^36^ ^37^

**Table 1:** Clinical and molecular characteristics of 21 Polish Leigh syndrome (LS) patients with *SURF1* gene mutations

Patient no.	Age at diagnosis (year of birth)	Clinical symptoms	Brain imaging, autopsy	Complex IV activity (% citric synthase)	Citric synthase activity (nmol/min/g protein)	Presence of c.841delCT SURF1 mutation at both alleles*/	
1	2 years (1987)	4 months: motor regression, floppiness, bulbar symptoms, hirsutism, lactic acidaemia		<3.0	70.4	c.841delCT	+
2	7 years (1987)	12 months: trembling, uncertain gait, myoclonic jerks, strabismus	MRI: hyperintensive signals at lenticular nuclei areas	5.7	146	c.841delCT	+
3	12 years (1987)	30 months: dystonic movements, failure to thrive	MRI: symmetric hyperintensive signals at lenticular and caudate nuclei, medulla oblongata putamen, globi pallidi	3.5	224.6	c.841delCT	+
4	3.5 years (1989)	5 months: failure to thrive, floppiness, nystagmus, irregular ventilation, trembling, hirsutism. LS diagnosed at autopsy of older brother		9.6	311	c.841delCT	c.841delCT
5	4 years (1989)	12 months: failure to thrive, vomiting, irregular ventilation. Death at the age of 4	CT: Symmetric hypodensive changes in basal ganglia	NA	NA	c.841delCT	c.841delCT
6	6.5 years (1990)	16 months: speech difficulties, hypotonia, nystagmus, motor regression. LS diagnosed at autopsy of sibling (see patient 11)		7.4	86	c.841delCT	+
7	3.75 years (1990)	2 years: difficulty in walking and speaking, failure to thrive, strabismus, hirsutism, hyperventilation episodes; CT of older brother: hypodense areas at LS typical localisation		8.4	121.7	c.841delCT	c.841delCT
8	3 years (1990)	12 months: failure to thrive, floppiness, hirsutism, irregular respiration. CT and autopsy of older sister revealed typical LS changes		NA	NA	c.841delCT	c.841delCT
9	3 years (1991)	14 months: regression of motor skills, failure to thrive, hirsutism, tremor, eye movement dissociation, apneic bouts, death at the age of 2.5 years		<3.0	175	c.841delCT	c.841delCT
10	2.5 years (1992)	3 months: failure to thrive, hypotonia, hirsutism; MRI of younger affected brother: LS changes in basal ganglia	CT: hypodensic areas in both cerebellar hemispheres and caudate nuclei	<3.0	136	c.841delCT	c.841delCT
11	10 years (1993)	16 months: nystagmus, speech and walking difficulties. Died at age of >10 years	At autopsy: spongiform lesions; vascular proliferation and neuronal loss in mesencephalon, diencephalon, medulla, and white matter of cerebellum	<3.0	104.1	c.841delCT	+
12	1.75 years (1994)	9 months: hypotonia, floppiness, failure to thrive, vomiting, tremor		NA	NA	c.841delCT	c.841delCT
13	2 years (1995)	19 months: tremor, eye movement dissociation, dystonia, irregular breathing	CT: Symmetric hypodense areas of basal ganglia	6.2	194.9	c.841delCT	c.841delCT
14	3 years (1996)	12 months; floppiness, irregular respiration, eye movement dissociation, strabismus, hirsutism	MRI at 2.5 years: symmetric hyperintensive signals in basal ganglia	3.7	111.4	c.841delCT	+
15	2.75 years (1997)	4 months: motor regression, failure to thrive, floppiness, tremor, hirsutism, lactic acidaemia		5.3	112.7	c.841delCT	c.841delCT
16	9 months (1997)	2 months: floppiness, vomiting, irregular respiration, eye dissociation, ptosis, hirsutism. LS in older brother	MRI at 4 years: symmetric hyperintensive signals in basal ganglia, brain atrophy	3.6	223	c.841delCT	c.841delCT
17	1.5 years (1997)	7 months; failure to thrive, vomiting, hypotonia, hirsutism, strabismus, lactic acidaemia		5.8	174	c.841delCT	c.841delCT
18	1.5 years (1998)	14 months: motor regression, trembling, hirsutism, hypotonia	MRI: symmetric hyperintensive signals in basal ganglia	4.3	133.2	c.841delCT	c.841delCT
19	1.5 years (2000)	7 months: motor regression, irregular respiration, vomiting	MRI: symmetric hyperintensive signals in basal ganglia	<3.0	296.6	c.841delCT	c.841delCT
20	1 year (2000)	In infancy: failure to thrive, vomiting, tremor, irregular ventilation, lactic acidaemia. Prolonged artificial ventilation before death	Autopsy at 4 years: Massive encephalomalacia obscurring topography of possible lesions. Severe mixed liver steatosis (90%); numerous lipid vacuoles in proximal convoloted renal tubules	<3.0	104.1	c.841delCT	+
21	1.5 years (2002)	6 months: failure to thrive, hypotonia, hyperventilation episodes, lactic acidaemia	MRI: symmetric hyperintensive signals at lenticular nuclei, putamen, crura cerebri, substantia nigra, cerebellum periventricular areas, medulla oblongata.	<3.0	209.7	c.841delCT	c.841delCT
Average (mean (SD))			4.7 (2.1)		175.7 (38.2)		
Reference (18 cases; mean (SD))			26.4 (7.4)		123.0 (26.0)		

CT, computer tomography; MRI, magnetic resonance imaging; NA, not analysed; +, presence of non-c.841delCT mutations.^13^ ^27^ ^36^

Skeletal muscle was obtained by open surgical biopsy of the vastus lateralis as a diagnostic procedure. Pathological studies comprised light microscopic assessment of frozen sections stained with haematoxylin and eosin (H&E), modified Gomori trichrome, oil red O, succinate dehydrogenase (SDH), COX, NADH dehydrogenase, acid phosphatase, and myosin ATPase at pH 4.3, 4.6, and 9.4. Assessment was performed in parallel with ongoing mitochondrial biochemical and clinical diagnostics.

For ultrastructural study the tissue was fixed in 2.5% cold glutaraldehyde for 1 h, washed in cacodylate buffer, post-fixed in 1% osmium tetroxide, dehydrated in graded alcohols and embedded in Epon 812 resin (DDSA, MNA; Serva, Heidelberg, Germany). The semi-thin sections were stained with toluidine blue to identify the region for ultrastructural study. Transverse and longitudinal sections were examined. Ultra-thin sections were counterstained with uranyl acetate and lead citrate and examined in a JEOL 1500 electron microscope (Tokyo, Japan). All muscle samples were processed for electron microscopy (EM) in the years 1994–2005.

The study protocol has been accepted by the institutional Childrens' Memorial Health Institute Bioethical Commission.

## RESULTS

Muscle light microscopy and electron microscopy results obtained from the individual patients are shown in the table 2.

**Table 2:** Histological, histochemical and electron microscopy findings in skeletal muscle of 21 children with Leigh syndrome (LS) and *SURF1* gene mutation

Patient no.	Age at biopsy (years)	COX deficiency	RRF	Lipid accumulation	Other pathology of skeletal muscle fibres	Abnormal MT*	Lipid droplets	Miofibrile loss	Cytoplasmic bodies	Concentric laminated bodies
1	2	++	−	+	Variability of diameter	+	−	−	−	−
2	7	++	−	++	Not found	+/−	+	+	−	−
3	12	++	−	++	Mild variability of diameter	−	+	+	−	−
4	3.5	NA	−	NA	Not found	+	−	+	−	−
5	4	NA	−	+	Not found	−	−	+	−	−
6	6.5	++	−	−	Variability of diameter	++	−	−	+	−
7	3.75	++	−	+	Not found	−	+	−	−	−
8	3	NA	−	−	Not found	NA	NA	NA	NA	NA
9	3	+	−	++	Not found	+	−	−	−	−
10	2.5	++	−	+	Mild variability of diameter	++	+	+	−	+
11	10	++	−	+	Variability diameter, fibre type grouping	−	+	−	−	−
12	1.75	+	−	+	Not found	+/−	+	+	−	−
13	2	++	−	++	Predominance of type I fibres	++	+	−	−	−
14	3	++	−	−	Mild interstitial fibrosis	+	+	−	−	−
15	2.75	++	−	+	Predominance of type I fibres	−	−	+	−	−
16	0.75	++	−	+	Predominance of type I fibres	++	+	−	−	−
17	1.5	+	−	++	Variability of diameter	+	−	+	−	−
18	1.5	+	−	++	Not found	+	+	−	−	−
19	1.5	+	−	++	Mild variability of diameter	−	+	−	−	−
20	1	++	−	−	Mild variability of diameter	−	−	−	−	−
21	1.5	+	−	++	Mild variability of diameter	NA	NA	NA	NA	NA

Light microscopy and electron microscope findings: +/− mild changes; + moderate changes; ++ severe changes; − no changes; *Mitochondrial (MT) ultrastructure abnormalities: +++: increase in mitochondrial number, changes in size, shape, and presence of electron dense inclusions; ++: increase in mitochondrial number, variation in size and shape; +: increase in mitochondrial number, slight variation in size and shape; +/−: slight increase in mitochondrial number.

COX, cytochrome c oxidase; NA, not analysed; RRF, ragged red fibre.

**Figure 1:**
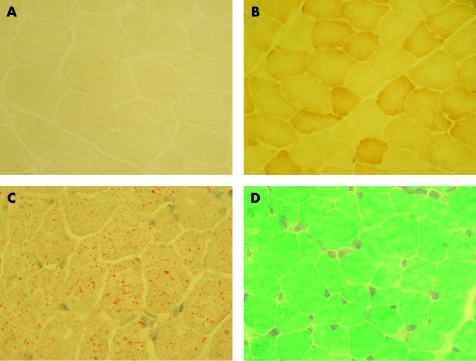
Histochemical and histological findings in the muscle of patients with Leigh syndrome associated with c. 841delCT *SURF1* gene mutation. A. Total diffuse cytochrome *c* oxidase (COX) deficit. B. Reference positive COX reaction (patient with encephalopathy of unknown cause examined in the same batch). C. Moderate lipid increase in muscle fibres. D. Variability of muscle fibre diameter.

Histological and histochemical pattern of muscle changes were similar in all children with *SURF1* gene mutations. Diffuse decreased activity or total deficit of COX activity was found in all patients (fig 1A,B).

In most children, COX deficit was accompanied by slight to moderate accumulation of lipids in muscle (fig 1C). Only three patients demonstrated no lipid increase. Half of the patients showed mild variability in muscle fibre diameter (fig 1D). Predominance of type 1 fibres was not found in the muscle with the exception of three cases. One case showed mild tendency for fibre type grouping. This phenomenon (not seen in any other patient) was considered unrelated to the primary molecular defect. No patient showed presence of RRF or increase of succinate dehydrogenase activity in muscle.

**Figure 2:**
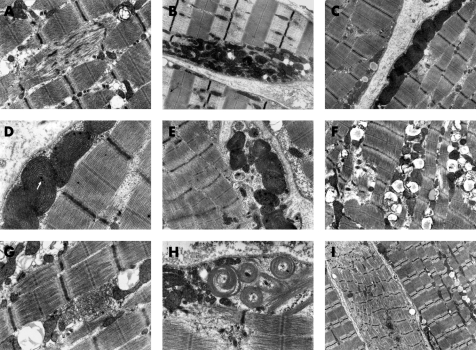
Ultastructural findings in the muscle of patients with Leigh syndrome associated with c. 841delCT *SURF1* gene mutation. In a majority of patients (12 of 19 examined cases), several muscle fibres demonstrated distinct subsarcolemmal accumulation of altered mitochondria (B, C). Frequently, the mitochondria were markedly enlarged or elongated (C, D) and exhibited dark matrix with densely packed, concentrically arranged lamellal cristae (D, E). Occasionally, the mitochondrial matrix displaced spaces with amorphous granular material and small, electron-dense, osmophilic granules (D). Extensive accumulation of lipid deposits occurred in association with normal and altered mitochondria (F, G). Some muscle fibres revealed alterations of myofibrils including their focal or widespread disorganisation and/or disruption (A, H, I). Moreover, the tubulofilamentous structures typical of cytoplasmic body formation and subsarcolemmal aggregation of concentric laminated bodies were also found in individual cases (H). Original magnification: A, ×12 000; B, C, F, ×7500; D, G, ×15 000; E, ×10 000; H, ×20 000; I, ×3000.

Ultrastructural examination of skeletal muscles biopsies revealed a spectrum of morphological abnormalities consisting mainly of more or less detectable mitochondrial alterations (fig 2). In a majority of patients (12 of 19 examined cases), several muscle fibres demonstrated distinct subsarcolemmal accumulation of altered mitochondria (fig 2B,C). Frequently, the mitochondria were markedly enlarged or elongated (fig 2C,D) and exhibited dark matrix with densely packed, concentrically arranged lamellar cristae (fig 2D,E). Occasionally, the mitochondrial matrix displaced spaces with amorphous granular material and small, electron-dense, osmophilic granules (fig 2D). Extensive accumulation of lipid deposits occurred in association with normal and altered mitochondria (fig 2F,G). Some muscle fibres revealed alterations of myofibrils including their focal or widespread disorganisation and/or disruption (fig 2A–H,I). Moreover, the tubulofilamentous structures typical of cytoplasmic body formation and subsarcolemmal aggregation of concentric laminated bodies were also found in individual cases (fig 2H).

**Figure 3:**
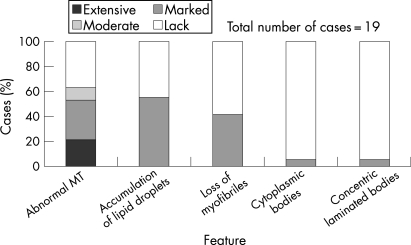
Distribution and frequency of various ultrastructural changes among 21 muscles of patients with SURF1-deficient Leigh syndrome.

Major changes in mitochondria on electron microscopy were not common in all SURF1-deficient LS patients, and were observed in a third of the cases (fig 3). The picture was normal in five cases and mild in two others.

No correlation was found between the electron microscopy findings and the light microscopy findings (ie, variability of fibre size), or with the clinical parameters (ie, age of biopsy and homozygous/heterozygous status of c.841delCT mutation).

A slightly significant correlation was found only between presence of lipid accumulation demonstrated by light and electron microscopy (Pearson test 0.39; p<0.05).

## DISCUSSION

SURF1-deficient LS presents a special sub-group of LS in which an as-yet unexplained homogeneity of changes in the brain^36^ ^37^ co-exist with a homogeneous pathogenetic molecular background. The exact role of the SURF1 protein has not been fully elucidated, but it is well known that without its contribution the COX complex is assembled improperly and functionally impaired.^38^^–^41 SURF1 acts in the early steps of COX assembly and may promote the association of the mitochondrially-encoded subunit COXII with the COXI–COXIV–COXV subassembly. There is a paucity of data on the typical morphological features of skeletal muscle in SURF1-deficient LS patients. In the majority of publications referring to *SURF1* gene mutations, skeletal muscle morphology is not included or it is limited to histochemical confirmation of COX deficit.^5^ ^12^ ^16^ ^19^ ^21^ ^22^–^24 26 28^ Only anecdotal reports about light microscopy findings in these 15 single case reports are available (table 3).

**Table 3:** Muscle histology and histochemistry of patients with *SURF1* gene mutation found in published case reports

Muscle histology and histochemistry	Age at biopsy	*SURF1* mutations	References
COX deficit, remarkable lipid accumulation; subsarcolemmal increase of NADH activity	5 years	A patient from Tirani’s SURF1-deficient complementary group with “atypical” LS	Tiranti *et al*,^3^ Tirani *et al*,^43^ Angelini *et al*^42^ and Munaro *et al*^44^
COX partial deficit, slight subsarcolemmal increase of SDH activity	Not reported	c.312del10insAT//c.567insCAGG	Sue *et al*^9^ and Van Coster *et al*^45^
Normal findings	1.5 years	c.589insCTGC//c.702C>T	Poyau *et al*^8^ and Collombet *et al*^46^
COX deficit, subsarcolemmal increase of SDH and NADH activity	4 years	c.312del10insAT//c.385G>A	Poyau *et al*^8^ and Collombet *et al*^46^
Normal findings (COX not assessed)	3 years	c.312del10insAT//c.385G>A	Poyau *et al*^8^ and Collombet *et al*^46^
Variability in fibre size	1.5 years	c.790delAG//c.820T>G	Teraoka *et al*^7^
COX deficit, lipid accumulation	2 years	c.240+1G>T// c.531_534delAAAT	Bruno *et al*^18^
COX deficit slight variability of fibre diameter, several hypotrophic fibres	5 years	Homozygous Q82X	Santoro *et al*^10^
Lipid accumulation, atrophy of type II fibres	2.5 years	Homozygous c.790_791delAG	Rahman *et al*^14^
COX deficit	<2 years	c.312del10insAT//c.688C>T	Tay *et al*^25^
COX deficit, type I fibres atrophy	<2 years	Homozygous c.834G>A	Tay *et al*^25^
COX deficit, mild non-specific changes	<2 years	As above (siblings)	Tay *et al*^25^
COX deficit; several RRFs	<2 years	c.312del10insAT//c 822_824dupTACAT	Tay *et al*^25^
COX deficit	Not done	c.608T>C//c.675_692del18	Sacconi *et al*^20^
Normal findings (including COX staining)*	3 years	Homozygous c.867G>A	Van Riesen *et al*^29^

*Severe isolated COX deficiency detected by biochemical assay.

COX, cytochrome c oxidase; LS, Leigh syndrome; RRF, ragged red fibre; SDH, succinate dehydrogenase.

The experimental model of SURF1-deficient mice presented with profound and isolated defect of COX activity, reduced histochemical reaction of COX, and mitochondrial proliferation.^47^

The cohort reported in this study was especially homogenous, carring the same c.841delCT mutation on both or one of alleles of the *SURF1* gene. This mutation is frequent among Polish, Czech and probably other Slavonic populations.^13^ ^15^ ^17^ ^27^ ^28^ ^36^ Here, we demonstrate that muscle biopsy of patients carrying the c.841delCT *SURF1* gene mutation show features that are similar to those found in the knock-out mice.^47^ In a manner akin to the results observed in the mouse model, all but two patients did not show a predominance of type I fibres (table 2). This is in agreement with the findings previously reported for cases with *SURF1* gene mutations (table 3). By contrast, this finding is considered frequent in the mitochondrial myopathies in general.^48^

**Take-home messages**

- SURF1-deficient skeletal muscle of patients with Leigh syndrome (LS) shows a reproducible and characteristic pattern of changes in light and electron microscopy that are potentially helpful in diagnosis.
- It should be kept in mind that those changes are not specific.
- Diffuse cytochrome c oxidase (COX) deficit remains the salient feature, and muscle may be interpreted as normal if the histochemical panel does not include COX reaction. The role of an experienced pathologist at this step of LS investigation is therefore important.

Among other minor features of the mitochondrial lesions lipid accumulation was seen more frequently in our material, and was present in 14 examined SURF1-LS specimens (table 2). Similarly the lipid accumulation was also commonly found in the SURF1-deficient LS muscles described earlier (table 3). The next quite frequent feature found in the studied material was variability of fibre size, present in nine muscle samples. Some degree of this abnormality was also mentioned in the previously reported cases (table 3). In general, the muscle morphology of these patients does not differ significantly from our findings. The so-called “Polish” *SURF1* mutation of c.841delCT, the most prevalent in our patients, was not found in any of the described cases.

It is important to emphasise that the presence of RRFs in the muscle, characteristic of mtDNA encoded mitochondrial myopathies, was extremely rare if not non-existent in the nuclearly encoded SURF1-LS muscle specimens, particularly in those carrying c.841delCT mutations. There is only one description of LS with *SURF1* mutations showing RRFs in a patient carrying two protein truncated mutations (table 3).^25^ RRFs were also not detected in the muscle of SURF1-deficient knock-out mice.^47^

However, the signs of increased mitochondria number seem not to be rare in the SURF1-deficient muscle. Subsarcolemmal NADH–tetrazolium positive rims,^42^ or intense subsarcolemmal SDH staining^45^ suggested incipient mitochondrial proliferation. Increased SDH reaction was also seen in the animal knock-out model of the disease.^47^ These reactions were negative in all our muscle specimens with homozygous and heterozygous c.841delCT *SURF1* gene mutation.

Ultrastructural assessment of knock-out mice revealed marked subsarcolemmal accumulation of enlarged mitochondria. Our ultrastructural findings in the affected human muscles were comparable to the animal model, also suggesting an increased number of mitochondria in several cases, but not in all of them. In the literature there is only scant information on the ultrastructure of human SURF1-deficient LS muscles. An exception is one case with COX-deficient Leigh syndrome reported in 1977.^49^ Thirty years later, a homozygous c.370C>A *SURF1* gene mutation was identified in this patient.^50^ The dominant ultrastructural feature of this case was enlarged mitochondria of bizarre shape and various size and form, aggregated in juxtanuclear and subsarcolemmal regions. The mitochondria contained an excessive number of irregularly arranged and distorted cristae. Fine granular electron-dense material was seen in the matrix, but paracristalline inclusions and large spherical dense bodies were absent. Myofibrils and myofilaments were preserved. Lipid droplets were seen in association with abnormal mitochondria.^49^

In agreement with this description, our ultrastructural study confirms that *SURF1* mutations may be associated with detectable structural manifestations of mitochondrial involvement. However, approximately a third of our SURF1-deficient muscle specimens (and the remaining two reports of ultrastructural findings in the literature)^7^ ^8^ did not demonstrate any ultrastructural anomalies of the mitochondria.

Ultrastructural abnormalities found by us were similar to these seen in the animal model of the disease.^47^ However it is not possible to identify any morphological or ultrastructural features that are exclusively and commonly present in muscles with *SURF1* mutations. Our results and those described in the literature data indicate that mitochondrial abnormalities can not be considered specific for the SURF1-deficient LS as it is unanimously accepted for all mitochondrial disorders, including those associated with mtDNA mutations.

On the basis of our study we propose that a diffuse deficit of COX in the muscle by histochemical staining, in particular in a child of Polish origin with a clinical phenotype of LS, should prompt direct molecular testing for the c.841delCT mutation in the *SURF1* gene. In our experience the contribition of an experienced clinical pathologist was very useful at this step of diagnosis of mitochondrial disorders in children.^51^ Additional histological features such as lack of RRFs and normal fibre size proportion, presence of lipid accumulation of mild to remarkable degree and fibre size variability had only limited diagnostic value in differential diagnosis of LS associated with *SURF1* gene mutations.

The electron microscopy showed no abnormalities in a third of our SURF1-deficient muscle biopsies, as in three of four descriptions found in the literature.^7^ ^8^ ^46^ This demonstrates that the diagnostic value of minor ultrastructural abnormalities is disputable, especially in children with a suspicion of mitochondrial disorder.^52^ ^53^

In conclusion, diffuse COX deficit in muscle biopsies is the only reproducible pathological finding of patients with LS. It should be emphasised that samples from LS patients solely assessed by means of microscopy or by histochemistry using a panel not including COX may be misinterpreted as normal.
